# Bioactive Potential of *Agave tequilana* Dry Juice Extract: Chemical Profiling, Antinociceptive Effects, and Synergistic Modulation with Diclofenac in the Formalin Test

**DOI:** 10.3390/ph19060863

**Published:** 2026-05-29

**Authors:** Reinner David Higuera-Quira, Juan Ramón Zapata-Morales, Josué Vidal Espinosa-Juárez, Elena Franco-Robles, Nereida Violeta Vega-Cabrera, Fidel Avila-Ramos, Clara Alba-Betancourt, Citlaly Natali de la Torre-Sosa, Osmar Antonio Jaramillo-Morales

**Affiliations:** 1 Maestría en Ciencias Farmacéuticas, Departamento de Farmacia, División de Ciencias Naturales y Exactas, Campus Guanajuato, Universidad de Guanajuato, Guanajuato 36200, Mexico; rd.higueraquira@ugto.mx (R.D.H.-Q.); juan.zapata@ugto.mx (J.R.Z.-M.); c.albabetancourt@ugto.mx (C.A.-B.); 2 Departamento de Farmacia, División de Ciencias Naturales y Exactas, Campus Guanajuato, Universidad de Guanajuato, Guanajuato 36200, Mexico; 3 Escuela de Ciencias Químicas, Universidad Autónoma de Chiapas, Ocozocoautla de Espinosa 29140, Mexico; josue.espinosa@unach.mx (J.V.E.-J.); citlaly.torre@unach.mx (C.N.d.l.T.-S.); 4 Departamento de Veterinaria y Zootecnia, División de Ciencias de la Vida, Campus Irapuato-Salamanca, Universidad de Guanajuato, Irapuato 36500, Mexico; e.franco@ugto.mx (E.F.-R.); ledifar@ugto.mx (F.A.-R.); 5 Departamento de Enfermería y Obstetricia, División de Ciencias de la Vida, Campus Irapuato-Salamanca, Universidad de Guanajuato, Irapuato 36500, Mexico; nv.vega@ugto.mx

**Keywords:** *Agave tequilana*, antinociceptive activity, synergism, diclofenac, molecular docking

## Abstract

**Background**: Safer analgesic strategies are needed to reduce the adverse effects associated with prolonged use of nonsteroidal anti-inflammatory drugs. The dry juice extract of Agave tequilana (ESPA), a chemically complex matrix with anti-inflammatory potential, may represent a promising adjuvant for modulating inflammatory pain. **Objective**: This study evaluated the antinociceptive activity of ESPA and its pharmacological interaction with diclofenac in the formalin test. **Methods**: BALB/c mice received ESPA or diclofenac orally 30 min before pain induction, and nociceptive behavior was quantified by counting paw flinches during the neurogenic and inflammatory phases. The GC–MS-detectable fraction of ESPA was chemically characterized, while the pharmacokinetic and bioactivity profiles of selected compounds were explored in silico using SwissADME and PASSonline. Molecular docking with COX-1 and COX-2 was performed using AutoDock Vina. Acute toxicity was evaluated according to OECD Guideline 423, and the ESPA–diclofenac interaction was examined using isobolographic analysis. **Results**: ESPA produced significant antinociceptive effects during the inflammatory phase. Although diclofenac exhibited greater potency, ESPA showed consistent efficacy in reducing inflammatory nociceptive behavior. GC–MS analysis identified several compounds within the detectable volatile/lipophilic fraction, including n-hexadecanoic, octadecanoic, and oleic acids. In silico evaluations suggested favorable predicted oral absorption and potential bioactivities related to inflammatory mediator regulation. Docking studies showed moderate predicted affinities for COX-1 and COX-2, lower than those observed for diclofenac. Isobolographic analysis demonstrated a synergistic interaction between ESPA and diclofenac, allowing dose reduction while maintaining antinociceptive efficacy. Acute toxicity testing indicated no signs of toxicity at the evaluated dose. **Conclusions**: These findings suggest that ESPA may act as a potential adjuvant in diclofenac-based analgesic strategies for inflammatory pain; however, further studies are required to clarify the active constituents and underlying mechanisms.

## 1. Introduction

Inflammatory pain is among the most prevalent and disabling clinical conditions worldwide. It is closely associated with tissue injury, immune activation, and the release of pro-inflammatory mediators, including interleukin-1β (IL-1β), IL-6, TNF-α, and prostaglandins [1,2,3]. Nonsteroidal anti-inflammatory drugs (NSAIDs), such as diclofenac, remain widely used as first-line pharmacological treatments because of their ability to inhibit cyclooxygenase isoforms (COX-1 and COX-2) and reduce prostaglandin synthesis [4,5]. However, prolonged NSAID use is limited by dose-dependent adverse effects involving the gastrointestinal tract, kidneys, and cardiovascular system. These safety concerns have encouraged the search for analgesic strategies that reduce NSAID exposure while preserving therapeutic efficacy [6].

Natural products have emerged as promising candidates for multitarget analgesic and anti-inflammatory strategies because of their complex chemical composition, potential to modulate immune and inflammatory responses, and generally favorable safety profiles [7,8]. Among them, Agave tequilana Weber var. azul, best known for its role in tequila production, has attracted increasing scientific interest due to the presence of biologically active constituents, including fructans, saponins, sterols, and long-chain fatty acids (LCFAs) [9,10]. Compounds and extracts from Agave species have been experimentally associated with anti-inflammatory activity and regulation of immune responses [11]. In particular, A. tequilana extracts have been shown to reduce cytokines such as IL-1β, IL-6, TNF-α, and IFN-γ and improve inflammatory outcomes in a pristane-induced murine model resembling systemic lupus erythematosus [12].

Long-chain fatty acids detected in the GC–MS profile of ESPA, including palmitic, stearic, and oleic acids, may contribute to anti-inflammatory responses through mechanisms reported for this class of compounds, such as modulation of eicosanoid pathways, changes in membrane lipid composition, and interaction with free fatty acid receptors, including FFA1/GPR40 and FFA4/GPR120 [12,13,14]. These pathways are involved in the regulation of cytokine release and nociceptor sensitization. However, because these mechanisms have been studied mainly in metabolic and immune contexts, their relevance to acute inflammatory pain and to the present experimental conditions should be interpreted cautiously and requires further investigation.

Despite accumulating evidence supporting the biological activity of A. tequilana-derived constituents, their antinociceptive potential remains poorly explored, particularly in combination with conventional analgesics. Such combinations may produce favorable pharmacological interactions, including synergistic effects, allowing dose reduction while preserving efficacy and potentially reducing NSAID-associated toxicity. This approach is consistent with current drug-development strategies that emphasize combination therapies to improve therapeutic outcomes [15].

Therefore, this study aimed to evaluate the antinociceptive activity of the dry juice extract of *Agave tequilana* (ESPA) and, more importantly, to characterize its pharmacological interaction with diclofenac using isobolographic analysis. In addition, the GC–MS-detectable fraction of the extract, the predicted pharmacokinetic properties of selected compounds, and their potential interactions with COX isoforms were explored to provide a preliminary mechanistic framework. This approach positions ESPA as a potential adjuvant in multimodal analgesic strategies rather than as a stand-alone therapeutic agent.

## 2. Results

### 2.1. The Gas Chromatography–Mass Spectrometry Analysis (GC–MS)

**Figure 1:**
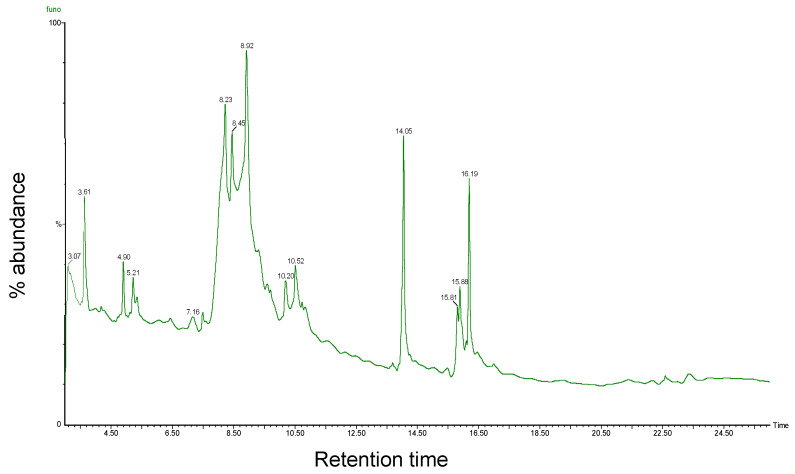
ESPA GC-MS chromatogram.

The GC–MS-detectable fraction of ESPA showed 12 identifiable compounds, as shown in Figure 1 and Table 1. These compounds exhibited a heterogeneous distribution within the detected volatile and semi-volatile profile, with long-chain fatty acids representing the most prominent group, followed by nitrogen-containing compounds, sugar-derived molecules, and aldehydes.

Compound identification was based on retention time and comparison of mass spectra with spectral libraries. The identified constituents, together with their corresponding retention times and relative peak areas, are shown in Table 1. Within the GC–MS-detectable fraction, n-hexadecanoic acid (palmitic acid) showed the highest relative peak area, representing 22.25% of the total chromatographic area and eluting at 14.05 min. Other relevant fatty acids were also identified, including octadecanoic acid (stearic acid; 10.83%) and oleic acid (8.01%), which eluted at 16.19 and 15.88 min, respectively. Together, these fatty acids accounted for a substantial proportion of the GC–MS chromatographic profile, explaining the higher-intensity peaks observed at longer retention times. Importantly, these compounds represent only the GC–MS-detectable lipophilic fraction of ESPA and should not be interpreted as the major constituents of the extract as a whole. ESPA has been previously characterized by HPLC with refractive index detection as being predominantly composed of agave fructans, mainly inulin (90.7%), along with minor amounts of simple sugars. Accordingly, high-molecular-weight fructans are not detected under the analytical conditions used in this GC–MS approach.

### 2.2. In Silico Studies

#### 2.2.1. Pharmacological Bioactivity and Pharmacokinetic Parameters (ADME)

The SwissADME platform was used to predict the pharmacokinetic and physicochemical properties of selected compounds detected in the GC–MS profile, specifically n-hexadecanoic acid, octadecanoic acid, and oleic acid (Table 2). These predictions were used as an exploratory approach to assess their potential oral bioavailability and selected parameters related to absorption, distribution, and metabolism.

All three compounds showed a low number of hydrogen bond donors (1) and acceptors (2), consistent with the low-polarity nature of long-chain fatty acids. Their predicted topological polar surface area values (TPSA = 37.30 Å^2^) were within a range generally considered favorable for membrane permeability, as TPSA values below 140 Å^2^ are commonly associated with good predicted oral absorption.

Regarding lipophilicity, the predicted MLOGP values for the three compounds were 5.55 for n-hexadecanoic acid, 6.33 for octadecanoic acid, and 6.11 for oleic acid, indicating high lipophilicity. This profile is consistent with the hydrophobic nature of long-chain fatty acids and may support membrane permeability through passive diffusion mechanisms.

In terms of aqueous solubility, the predicted log S (ESOL) values ranged from −5.02 to −5.73, indicating moderate to low solubility, as expected for highly lipophilic compounds. According to SwissADME, the three compounds showed high predicted gastrointestinal absorption, suggesting a potentially favorable oral absorption profile despite their elevated lipophilicity. All three compounds complied with Lipinski’s Rule of Five, with only one violation related to high log *p* values (>5). A single violation does not necessarily preclude drug-likeness; however, these results should be interpreted as preliminary physicochemical predictions rather than evidence of pharmacological efficacy. No PAINS (Pan-Assay Interference Compounds) alerts were detected for any of the evaluated compounds, suggesting a low probability of nonspecific assay interference.

The SwissADME predictions also suggested that n-hexadecanoic acid and oleic acid may inhibit CYP2C9, whereas octadecanoic acid was not predicted to inhibit this enzyme. This potential interaction with CYP2C9 should be interpreted cautiously, as it is based on an in silico prediction. Nevertheless, it may be relevant for future safety assessments, particularly in studies evaluating possible pharmacokinetic interactions with co-administered drugs such as diclofenac.

The PASSonline platform was used to predict the possible pharmacological activities of the main compounds identified in the extract, based on their chemical structure. The results are expressed as probability of activity (Pa) and probability of inactivity (Pi), where Pa values > 0.7 and low Pi values indicate a high likelihood that the compound exhibits the predicted biological activity (Table 3).

PASSonline predicted putative biological activities related to inflammatory medi-ator regulation for n-hexadecanoic acid, octadecanoic acid, and oleic acid, including ac-tivities associated with prostaglandin, leukotriene, and thromboxane pathways. These predictions were used as an exploratory bioactivity screening approach.

Molecular docking analysis was performed to explore the predicted binding affinities and interaction modes of selected GC–MS-detectable fatty acids, namely n-hexadecanoic acid, octadecanoic acid, and oleic acid, with cyclooxygenase-1 (COX-1) and cyclooxygenase-2 (COX-2). Diclofenac was included as a reference drug for comparative purposes (Table 4). The analyzed parameters included predicted binding affinity, interaction type, and the amino acid residues involved in ligand binding within the active site.

#### 2.2.2. Molecular Docking

**Figure 2:**
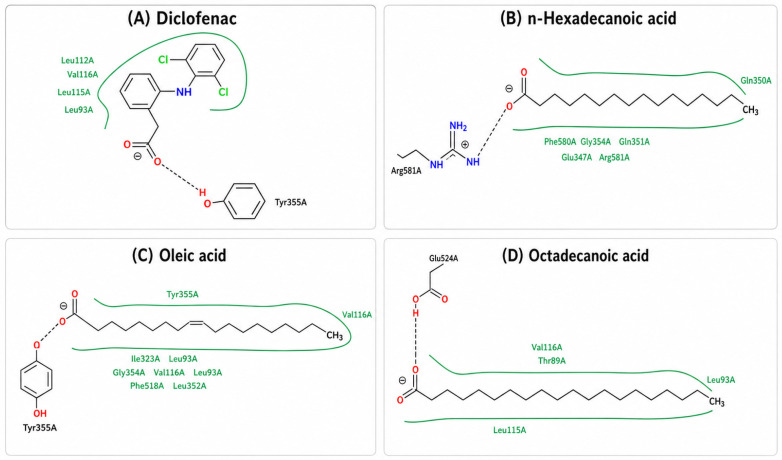
Interactions between the COX-1 receptor protein and selected compounds identified through in silico molecular docking: (**A**) diclofenac, (**B**) n-hexadecanoic acid, (**C**) oleic acid, and (**D**) octadecanoic acid. Dashed black lines indicate hydrogen-bond interactions. Green curved lines indicate hydrophobic contact regions. Residues involved in ligand interactions are shown in green. Oxygen and nitrogen atoms are shown in red and blue, respectively.

**Figure 3:**
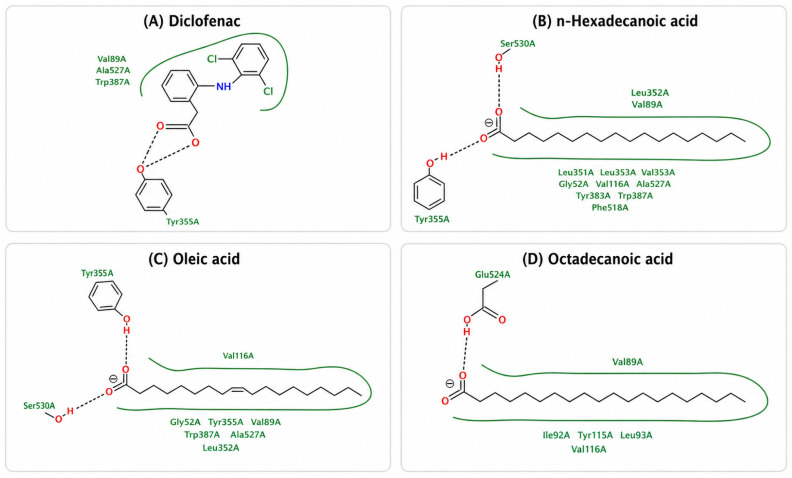
Interactions between the COX-2 receptor protein and selected compounds identified through in silico molecular docking: (**A**) diclofenac, (**B**) n-hexadecanoic acid, (**C**) oleic acid, and (**D**) octadecanoic acid. Dashed black lines indicate hydrogen-bond interactions. Green curved lines indicate hydrophobic contact regions. Residues involved in ligand interactions are shown in green. Oxygen and nitrogen atoms are shown in red and blue, respectively.

n-Hexadecanoic acid showed similar predicted binding affinities for both enzymes (−6.116 kcal/mol for COX-1 and −6.123 kcal/mol for COX-2), with estimated inhibition constants close to 32 µM. These values suggest comparable, nonselective predicted affinity toward both COX isoforms. As shown in Table 4 and Figure 2 and Figure 3, n-hexadecanoic acid displayed an ionic interaction with Arg581A in COX-1, along with hydrophobic interactions involving residues such as Phe580A, Gly354A, Gln351A, and Glu347A. In COX-2, this ligand formed two hydrogen bonds and several hydrophobic contacts with residues within the catalytic channel, including Ser530A, Tyr385A, Tyr355A, and Trp387A. Octadecanoic acid showed a slightly higher predicted affinity for COX-1 (−6.296 kcal/mol) than for COX-2 (−5.021 kcal/mol), suggesting a possible preference for COX-1 in this docking model. The interactions observed included one hydrogen bond and multiple hydrophobic interactions with residues such as Val116A, Leu115A, and Leu93A in COX-1. In COX-2, the interactions were weaker and fewer in number, which is reflected in the higher Ki value (Table 4, Figure 2 and Figure 3).

Oleic acid showed predicted binding affinities of −5.698 kcal/mol for COX-1 and −5.370 kcal/mol for COX-2 (Table 4; Figure 2 and Figure 3), indicating moderate to low affinity compared with the other evaluated fatty acids. In COX-1, this ligand showed ionic interactions with arginine residues and multiple hydrophobic contacts. In COX-2, oleic acid formed two hydrogen bonds and hydrophobic contacts with residues located within the active site, including Ser530A, Tyr385A, and Tyr355A. Overall, the predicted ΔG values obtained for the evaluated fatty acids ranged from −5.021 to −6.296 kcal/mol, suggesting moderate predicted affinity toward both COX isoforms. In comparison, diclofenac showed stronger predicted affinity, particularly for COX-2 (−8.202 kcal/mol), consistent with its known pharmacological profile as an NSAID.

### 2.3. In Vivo Studies

#### 2.3.1. Acute Toxicity Evaluation

In the acute oral toxicity study, oral administration of ESPA at the limit dose of 2000 mg/kg did not produce mortality during the 14-day observation period. No clinical signs of toxicity were observed. Treated animals did not show behavioral alterations suggestive of intoxication, including changes in locomotor activity, posture, response to stimuli, or general condition. Body weight remained stable throughout the experimental period. After euthanasia and macroscopic examination, no visible alterations were detected in the evaluated systems, including the skin, mucous membranes, respiratory and cardiovascular systems, central nervous system, and somatomotor function. No lesions or macroscopic changes compatible with acute toxicity were observed. According to the criteria established in OECD Guideline 423, the absence of mortality at 2000 mg/kg indicates an LD50 greater than 2000 mg/kg, corresponding to Category 5 or “unclassified” under the acute oral toxicity classification system.

#### 2.3.2. Effect of ESPA and Diclofenac on Nociceptive Behavior in the Formalin-Induced Pain Model

**Figure 4:**
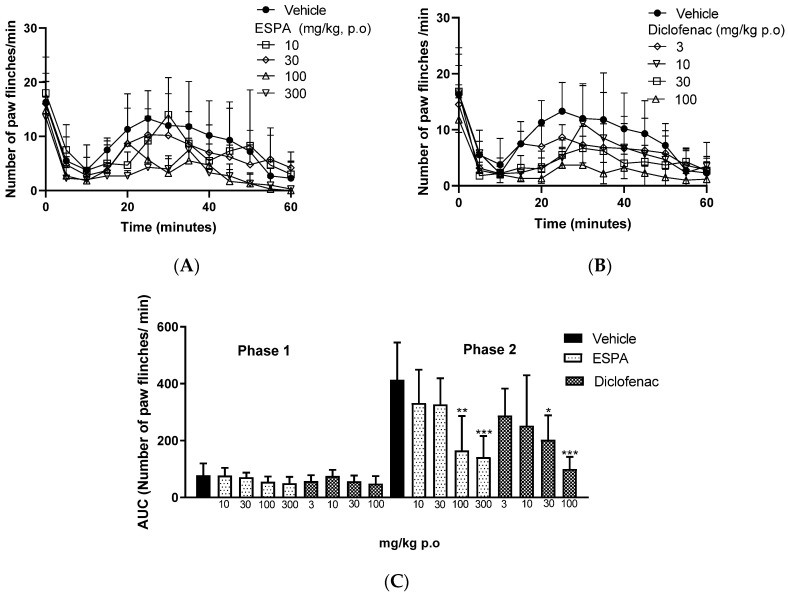
(**A**) Time course of the number of flinches in mice treated with ESPA (10, 30, 100, and 300 mg/kg, p.o.) prior to the injection of 2% formalin and (**B**) mice treated with diclofenac (3, 10, 30, and 100 mg/kg, p.o.). (**C**) Area under the curve (AUC) of the number of flinches during phase I and phase II in mice treated with ESPA (10, 30, 100, and 300 mg/kg, p.o.) or the reference drug diclofenac (3, 10, 30, and 100 mg/kg, p.o.) prior to the injection of 2% formalin. Animals in the control group received vehicle (saline solution). Data are expressed as mean ± SD (*n* = 6 per group). * *p* < 0.05, ** *p* < 0.01, *** *p* < 0.001 vs. vehicle (one-way ANOVA followed by Dunnett’s post hoc test).

Subcutaneous administration of 2% formalin into the dorsal surface of the right hind paw produced the expected biphasic nociceptive behavior. An immediate nociceptive response was observed during phase I (0–10 min), followed by a second response during phase II (15–60 min), which is predominantly associated with inflammatory pain. Figure 4A shows the time course of paw flinches in mice treated orally with ESPA (10, 30, 100, and 300 mg/kg) 30 min before 2% formalin injection. ESPA reduced the number of flinches in a dose-related manner compared with the vehicle group, with the greatest reduction observed at 300 mg/kg. Figure 4B shows the time course of paw flinches in mice treated orally with diclofenac (3, 10, 30, and 100 mg/kg) 30 min before 2% formalin injection. Diclofenac also reduced the number of flinches in a dose-related manner compared with the vehicle group, with the greatest reduction observed at 100 mg/kg.

Based on the time-course curves, the area under the curve (AUC) was calculated for each phase using the trapezoidal method. Figure 4C shows the AUC values for phase I (neurogenic phase) and phase II (inflammatory phase) in mice treated orally with ESPA (10, 30, 100, and 300 mg/kg) or diclofenac (3, 10, 30, and 100 mg/kg). AUC analysis showed that neither ESPA nor diclofenac produced significant differences compared with the vehicle group during phase I (F(8, 45) = 1.3, *p* = 0.231, η^2^ = 0.19; Figure 4C). In contrast, both ESPA and diclofenac significantly reduced nociceptive behavior during phase II compared with the vehicle group (F(8, 45) = 5.1, *p* < 0.001, η^2^ = 0.48; Figure 4C).

**Figure 5:**
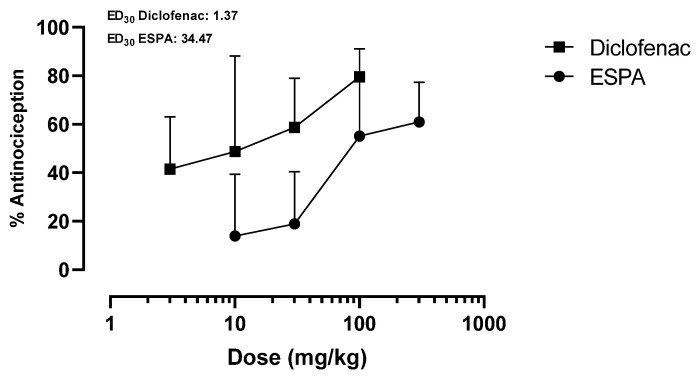
Dose–response curve for the antinociceptive effect of ESPA (10, 30, 100, and 300 mg/kg, p.o.) and the reference drug diclofenac (3, 10, 30, and 100 mg/kg, p.o.) administered prior to the injection of 2% formalin. Data are expressed as mean ± SD (*n* = 6 per group).

Dose–response analysis showed that both ESPA and diclofenac produced dose-related increases in antinociceptive effect after 2% formalin administration (Figure 5). Under these experimental conditions, ESPA showed lower efficacy than diclofenac (*p* < 0.05). The ED30 value was 34.47 mg/kg for ESPA and 1.37 mg/kg for diclofenac. These values indicate that diclofenac exhibited greater potency than ESPA, as a lower dose was required to produce the same level of effect.

#### 2.3.3. Effect of the Combination of ESPA and Diclofenac on Nociceptive Behavior in the Formalin-Induced Pain Model

Table 5 shows the fixed-ratio combinations of diclofenac and ESPA, including the individual doses of each component and the total dose administered for each combination. These doses were calculated based on the ED30 values obtained for each treatment administered alone.

**Figure 6:**
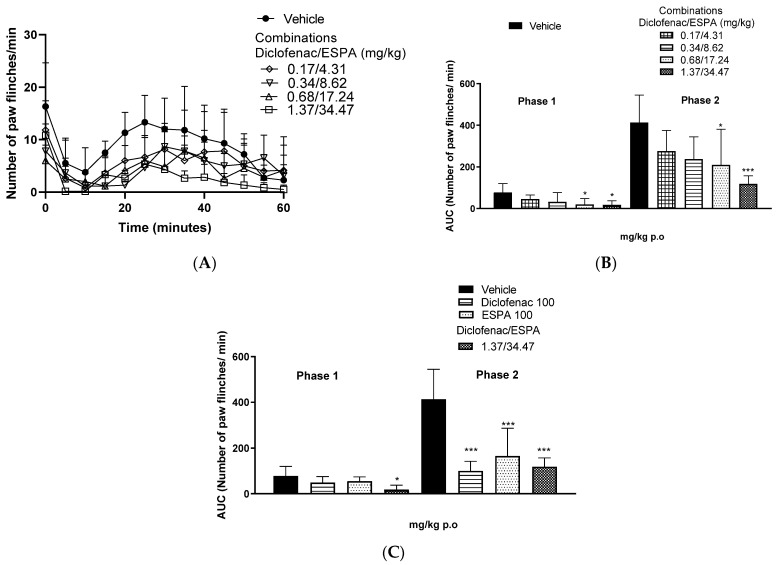
(**A**) Time course of the number of flinches in mice treated with different combinations of diclofenac plus ESPA administered prior to the injection of 2% formalin. (**B**) Area under the curve (AUC) of the number of flinches during phase I and phase II of the formalin test in mice treated with combinations of diclofenac plus ESPA, administered orally prior to the injection of 2% formalin. (**C**) Area under the curve (AUC) of the number of flinches during phase I and phase II in mice treated with the diclofenac/ESPA combination at a dose of 1.37/34.47 mg/kg, respectively, compared with the effect produced by individual administration of diclofenac or ESPA at a dose of 100 mg/kg (p.o.) prior to the injection of 2% formalin. Control animals received vehicle (saline solution). Data are expressed as mean ± SD (*n* = 6 per group). * *p* < 0.05, *** *p* < 0.001 vs. vehicle (one-way ANOVA followed by Dunnett’s post hoc test).

Figure 6A shows the time course of paw flinches induced by 2% formalin in mice pretreated with different fixed-ratio combinations of ESPA and diclofenac over the 60 min observation period. The vehicle-treated group showed a sustained nociceptive response throughout the observation period. In contrast, mice treated with ESPA–diclofenac combinations showed a reduction in the number of paw flinches compared with the vehicle group.

The reduction in nociceptive behavior followed a dose-related pattern, with higher doses of the ESPA–diclofenac combinations producing greater decreases in paw flinches compared with the vehicle group. Among the evaluated combinations, diclofenac/ESPA at 1.37/34.47 mg/kg produced the largest reduction in flinches over time within the tested dose range. The area under the curve (AUC) for nociceptive behavior was calculated for all experimental groups using the trapezoidal method. Figure 6B shows the AUC values for phase I (neurogenic phase) and phase II (inflammatory phase) of the formalin test in mice treated orally with the different diclofenac/ESPA combinations.

AUC analysis showed that during phase I (F(4, 25) = 3.4, *p* < 0.05, η^2^ = 0.35) and II (F(4, 25) = 5.0, *p*< 0.05, η^2^ = 0.45), the diclofenac/ESPA combinations at 0.68/17.24 and 1.37/34.47 mg/kg significantly reduced nociceptive behavior compared with the vehicle group (Figure 6B). A direct comparison was also performed between the diclofenac/ESPA combination at 1.37/34.47 mg/kg and the individual administration of diclofenac or ESPA at 100 mg/kg (Figure 6C).

AUC analysis showed that the diclofenac/ESPA combination at 1.37/34.47 mg/kg produced statistically significant differences compared with the vehicle group during phase I (F(3, 20) = 4.581, *p* < 0.05, η^2^ = 0.41). During phase II, the same combination produced a reduction in nociceptive behavior comparable to that observed following individual administration of diclofenac or ESPA at 100 mg/kg.

All evaluated treatments, including the diclofenac/ESPA combination and the individual doses of diclofenac and ESPA, showed statistically significant differences during phase II compared with the vehicle group (F(3, 20) = 14.50, *p* < 0.001, η^2^ = 0.68).

#### 2.3.4. Isobolographic Analysis of the Antinociceptive Interaction Between the ESPA–Diclofenac Combination

**Figure 7:**
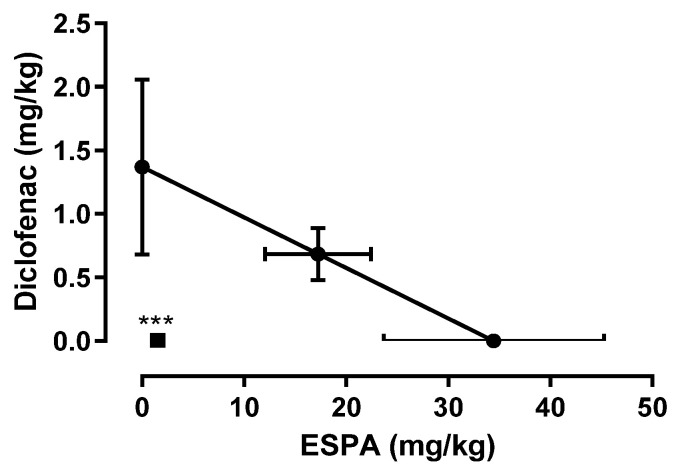
Isobologram analysis of the pharmacological interaction between diclofenac and ESPA administered orally in the formalin-induced pain model. Horizontal and vertical bars represent the SD. The oblique line connecting the X- and Y-axes represents the theoretical additivity line. The midpoint of this line corresponds to the theoretical additive point calculated from the ED_30_ values of each drug administered alone. Experimental points located below the additivity line indicate a synergistic interaction. Data are expressed as mean ± SD. (*n* = 6). *** *p* < 0.001 vs. theoretical additive point (Student’s *t*-test).

To characterize the pharmacological interaction between diclofenac and ESPA, an isobologram was constructed using the ED30 values obtained for each treatment administered alone and for their fixed-ratio (1:1) combinations (Figure 7). In the isobologram, the *x*-axis represents the ED30 of ESPA (34.47 ± 10.47 mg/kg), whereas the *y*-axis represents the ED30 of diclofenac (1.37 ± 0.69 mg/kg). The line connecting these values defines the theoretical additivity line, and its midpoint corresponds to the expected additive dose in the absence of pharmacological interaction. The experimental ED30 of the diclofenac–ESPA combination was located below the theoretical additivity line. The experimental ED30 for the combination was 1.57 ± 0.66 mg/kg, which was significantly lower (*p* < 0.001) than the theoretical additive ED30 of 17.92 ± 5.40 mg/kg. The interaction index (γ) was 0.087 ± 0.04.

## 3. Discussion

The present study evaluated the antinociceptive activity of ESPA, its pharmacological interaction with diclofenac, and potential mechanisms involved by integrating in vivo pharmacological assays, exploratory in silico analyses, pharmacokinetic predictions, and acute toxicity assessment.

The formalin test showed that both ESPA and diclofenac significantly reduced nociceptive behavior during phase II, which is predominantly associated with inflammatory pain. This pattern is consistent with the pharmacological profile of NSAIDs, which primarily reduce inflammatory nociception through inhibition of prostaglandin synthesis mediated by cyclooxygenase activity [16]. The antinociceptive effect observed with ESPA is also consistent with previous studies reporting analgesic and anti-inflammatory activity in other Agave species and in natural extracts containing saponins and other bioactive constituents.

For example, *Agave sisalana* extracts have been reported to inhibit formalin-induced nociceptive behavior during the inflammatory phase at doses of 25 mg/kg [17]. Similarly, Monterrosas-Brisson et al. (2012) [18] reported that extracts from several *Agave* species, including *A. tequilana*, *A. angustifolia*, and *A. americana*, exerted anti-inflammatory effects in experimental models, supporting the pharmacological relevance of *Agave*-derived constituents in inflammation-associated responses. In addition, hydrolyzed extracts of *Agave sisalana* showed significant antinociceptive and anti-inflammatory effects in carrageenan-induced edema and acetic acid-induced nociception, with activity comparable to acetylsalicylic acid [19]. Finally, saponin-enriched fractions from *Agave brittoniana* exhibited anti-inflammatory activity in both acute and chronic inflammatory models, supporting the relevance of Agave-derived constituents in inflammatory modulation [20].

Although the ED30 of ESPA (34.47 ± 10.78 mg/kg) was higher than that of diclofenac (1.37 ± 0.69 mg/kg), the extract produced a consistent antinociceptive effect during the inflammatory phase. This effect is comparable to that reported for other plant-derived preparations with activity on inflammatory responses [21,22].

In this context, the long-chain fatty acids (LCFAs) detected in the GC–MS profile of ESPA, particularly n-hexadecanoic acid, octadecanoic acid, and oleic acid, may represent plausible contributors to the biological activity of the extract. These compounds have been associated with anti-inflammatory properties in previous studies. Calder (2012) [23] reported that fatty acids can alter the phospholipid composition of immune cell membranes, thereby influencing inflammatory eicosanoid formation, cell signaling, and NF-κB-regulated gene expression. Recent studies also suggest that certain LCFAs can interact with free fatty acid receptors, including FFA1/GPR40 and FFA4/GPR120, in immune cells such as macrophages and neutrophils. These pathways are involved in the regulation of cytokine release, including IL-6, IL-1β, and TNF-α, and may influence peripheral nociceptive sensitization [24]. In addition, long-chain polyunsaturated fatty acids may serve as precursors of specialized pro-resolving mediators, such as resolvins and protectins, which participate in inflammation resolution and tissue homeostasis [25,26].

In addition, the inulin- and agavin-type fructans present in ESPA may contribute to the overall biological profile of the extract through anti-inflammatory and immune-regulatory properties reported for Agave-derived carbohydrates [27,28]. However, given the acute experimental design used in the present study, their contribution to the observed antinociceptive effect should be interpreted with caution. Under these conditions, the effect of ESPA is more likely associated with rapid modulation of peripheral inflammatory signaling than with delayed mechanisms requiring prolonged exposure.

Evidence from previous studies also indicates that fructans from Agave tequilana can reduce oxidative stress and NF-κB activation under experimental conditions involving longer exposure periods, supporting their potential involvement in inflammatory response regulation [22]. Thus, ESPA should be interpreted as a chemically complex matrix whose biological effects may arise from the combined contribution of fructans, saponins, and low-molecular-weight lipophilic constituents, rather than from a single identified compound.

From a computational perspective, PASSonline predictions suggested that selected GC–MS-detectable compounds in ESPA, namely n-hexadecanoic acid, oleic acid, and octadecanoic acid, may be associated with biological activities related to inflammatory mediator regulation, including pathways involving prostaglandins, leukotrienes, thromboxanes, and cytokines. However, these predictions were used only as an exploratory bioactivity screening approach and should not be interpreted as direct evidence of target modulation.

Molecular docking was focused on COX-1 and COX-2 because these enzymes are directly involved in prostaglandin biosynthesis and represent the main pharmacological targets of diclofenac, the reference NSAID used in this study. Thus, docking analysis was intended to provide a focused comparison with diclofenac rather than a comprehensive validation of all PASSonline-predicted targets. The docking results suggested possible interactions between GC–MS-detectable long-chain fatty acids and COX isoforms. However, the predicted binding affinities were moderate and lower than those observed for diclofenac, and direct experimental validation of COX inhibition was not performed. Therefore, these findings should be considered preliminary and hypothesis-generating rather than confirmatory. Additional predicted targets should be evaluated in future studies using complementary computational approaches, enzymatic assays, and inflammatory mediator measurements.

In addition, SwissADME analyses showed that these metabolites have high predicted gastrointestinal absorption, no PAINS alerts, and favorable permeability-related properties, supporting their potential oral bioavailability.

It is important to emphasize that the computational analyses performed in this study, including PASS predictions and molecular docking, were intended exclusively as exploratory tools to support hypothesis generation. These approaches do not provide direct experimental evidence of target modulation or enzyme inhibition. In the absence of biochemical validation, such as COX inhibition assays or inflammatory mediator quantification, the proposed mechanistic interpretations remain speculative and should be interpreted with caution.

Accordingly, the biological effects observed in vivo cannot be conclusively attributed to specific molecular targets based on computational results alone, and further experimental studies are required to validate these predictions.

The isobolographic analysis demonstrated a synergistic interaction between ESPA and diclofenac, as the experimental ED30 of the combination was markedly lower than the theoretical additive dose. This finding is consistent with previous studies reporting synergistic interactions between NSAIDs and natural products with complementary pharmacological profiles [29,30]. The observed synergy suggests that diclofenac exposure may be reduced while maintaining antinociceptive efficacy, which is relevant considering the gastrointestinal, cardiovascular, and renal adverse effects associated with prolonged NSAID use [31]. This interaction may be explained by a complementary pharmacological model in which diclofenac provides potent COX inhibition, whereas ESPA may contribute to the modulation of inflammatory pathways through its chemically diverse composition, including fructans and GC–MS-detectable long-chain fatty acids. Such complementary actions may enhance the overall antinociceptive effect beyond simple additivity.

Importantly, although previous studies have documented the anti-inflammatory and analgesic properties of Agave species [17,18,19,20,21,32], the present work provides a distinct contribution. The main novelty of this study does not lie solely in demonstrating the intrinsic biological activity of ESPA, but rather in quantitatively evaluating its pharmacological interaction with diclofenac. In this context, isobolographic analysis demonstrated a synergistic interaction, supporting a potential NSAID dose-sparing effect. This distinction is particularly relevant because it positions ESPA not as a stand-alone analgesic, but as a potential adjuvant in multimodal analgesic strategies aimed at maintaining efficacy while reducing adverse effects associated with conventional therapies [29,30].

The acute oral toxicity study showed that ESPA produced no mortality or observable signs of toxicity at 2000 mg/kg, corresponding to Category 5 or “unclassified” according to OECD Guideline 423. This finding is consistent with low acute oral toxicity under the experimental conditions evaluated. However, because only acute toxicity was assessed, further studies addressing repeated-dose toxicity, pharmacokinetic behavior, and long-term safety are required before broader pharmacological or translational conclusions can be drawn.

A critical aspect that should be emphasized is the limited phytochemical resolution of the current study. Although GC–MS analysis enabled the identification of several volatile and semi-volatile constituents, this approach does not capture the predominant fraction of the extract, which is mainly composed of inulin-type fructans (~90.7%) and other non-volatile carbohydrates. Therefore, the detected long-chain fatty acids and minor compounds should not be interpreted as the principal constituents of ESPA, nor can they be directly linked to the observed pharmacological effects.

Consequently, the relationship between chemical composition and biological activity remains only partially understood. The antinociceptive and synergistic effects observed in vivo should be attributed to the extract as a whole rather than to specific identified compounds. Any discussion regarding the contribution of individual constituents, including fatty acids or minor phytochemicals, should be considered hypothetical and supported primarily by indirect evidence.

Despite the strengths of the experimental design, the scope of the present study should be interpreted within certain methodological considerations. The antinociceptive activity of ESPA was evaluated exclusively using the formalin-induced pain model, which primarily reflects acute inflammatory nociception. Although this model is well established, it does not fully represent other clinically relevant pain conditions, such as neuropathic, visceral, or chronic inflammatory pain, thereby limiting the extrapolation of the findings to broader pain contexts.

The mechanistic interpretation should also be considered preliminary. Although isobolographic analysis demonstrated a synergistic interaction between ESPA and diclofenac, the underlying molecular mechanisms were inferred mainly from exploratory in silico predictions, docking analyses, and indirect evidence. Direct biochemical validation, such as measurement of inflammatory mediators, enzyme activity assays, and pharmacokinetic analyses, was not performed. Therefore, future studies should confirm the proposed mechanisms and clarify the systemic exposure of ESPA-derived constituents.

The phytochemical characterization was inherently partial. Although GC–MS allowed the detection of several volatile and semi-volatile constituents, including long-chain fatty acids such as n-hexadecanoic acid, octadecanoic acid, and oleic acid, this approach does not provide a comprehensive chemical profile of ESPA. In particular, polar, thermolabile, and high-molecular-weight constituents, including fructans and other carbohydrate-derived metabolites, are not adequately characterized by this method. Therefore, the present data do not allow definitive attribution of the antinociceptive activity or the synergistic interaction with diclofenac to specific individual compounds. The identified fatty acids should be interpreted as plausible contributors within a complex phytochemical matrix rather than as confirmed active principles. Future studies should include LC–MS/MS-based metabolomic profiling, chromatographic fractionation, bioassay-guided isolation, and structural elucidation to define the chemical entities responsible for the observed pharmacological effects.

Finally, because ESPA is a commercially processed extract, detailed information regarding plant material origin, the specific plant part used, processing conditions, and batch-level standardization parameters is limited. Although the product is described by the manufacturer as a standardized Agave tequilana dry juice extract rich in inulin-type fructans, the limited transparency in production and quality control parameters may affect reproducibility across independent studies. Further studies using well-characterized and fully traceable plant material, as well as standardized extracts with defined chemical markers, are necessary to improve reproducibility and facilitate mechanistic interpretation.

An additional limitation is related to the use of a commercially processed extract, which inherently restricts pharmacognostic characterization and experimental reproducibility. Although the product is described as a standardized extract rich in inulin-type fructans, detailed information regarding batch-to-batch variability, raw material sourcing, and processing conditions is not fully available. The use of the extract “as received” without further purification or chemical standardization may introduce variability that cannot be controlled within the current experimental framework.

This limitation should be considered when interpreting the pharmacological findings, as reproducibility across independent studies may depend on factors not fully characterized here. Future research should incorporate well-defined plant material, standardized extraction procedures, and the use of chemical markers to ensure consistency and improve translational relevance.

## 4. Materials and Methods

### 4.1. Compounds

A 37% formaldehyde solution was obtained from J.T. Baker (Easton, Pennsylvania, USA. For the formalin test, a 2% formaldehyde solution was prepared in distilled water and administered subcutaneously into the right hind paw in a volume of 20 μL using a 30-gauge needle. ESPA was commercially obtained from Grupo SOLAVE (Amatitán, Jalisco, Mexico). The agave-derived product had been previously characterized by HPLC with refractive index (RI) detection using an Agilent 1260 system. According to the manufacturer, the extract is derived from *Agave tequilana Weber var. azul* and consists primarily of inulin-type fructans. ESPA is a white powder consisting of a polydisperse mixture of fructans with different degrees of polymerization; its composition includes 90.7% inulin, 5.1% fructose, 2.0% glucose, 1.8% sucrose, and 0.4% other carbohydrates. The extract was used as received, without further purification. The drug used was diclofenac, obtained from PiSA (Mexico, Laboratories, Mexico City, Mexico). Diclofenac and ESPA were suspended in 0.9% saline solution (*w*/*v*) and administered orally at a volume of 1 mL/kg. All substances were freshly prepared before each use.

### 4.2. Gas Chromatography–Mass Spectrometry (GC–MS)

For GC–MS analysis, ESPA analytes were extracted using ultrasound-assisted extraction. Briefly, 2 g of ESPA were placed in a 50 mL beaker, and 20 mL of HPLC-grade methanol (J.T. Baker) were added. The mixture was sonicated for 2 min using an ultrasonic processor (Cole-Parmer Instrument Company, Vernon Hills, IL, USA) equipped with a 1/8-inch conical tip, operating at 500 W and a 20% duty cycle. The methanolic extract was separated by decantation, and the extraction procedure was repeated two additional times with 20 mL of fresh methanol. The combined methanolic extracts were concentrated using a rotary evaporator at 45 °C to a final volume of approximately 2 mL. The concentrated extract was transferred to a 2 mL amber vial and stored at −20 °C until GC–MS analysis.

No derivatization step was performed prior to GC–MS analysis. Therefore, the chromatographic profile should be interpreted as representing the volatile and semi-volatile GC–MS-detectable fraction of ESPA under the analytical conditions used. High-molecular-weight carbohydrates, including inulin-type fructans, are not detectable using this GC–MS workflow.

The GC–MS-detectable fraction of ESPA was analyzed using a PerkinElmer 560S mass spectrometer (PerkinElmer Inc., Waltham, MA, USA) coupled to an Elite-5MS capillary column (30 m × 0.25 mm × 0.25 µm film thickness; PerkinElmer Inc., Waltham, MA, USA). The initial oven temperature was held at 80 °C for 1 min, increased at 10 °C/min to 220 °C and held for 6 min, and then increased at 20 °C/min to 290 °C, where it was maintained for an additional 6 min. The injector and transfer line temperatures were set at 180 °C and 220 °C, respectively. Helium was used as the carrier gas at a constant flow rate of 1 mL/min.

The solvent delay was set to 3 min, and 1 µL of the concentrated methanolic extract was injected automatically in split mode using the autosampler. Mass spectra were acquired by electron ionization (EI) at 70 eV over an *m*/*z* range of 35–580 in full-scan mode. The ion source temperature was set at 200 °C. Compounds were tentatively identified by comparing their retention times and mass spectra with those available in the NIST 11 spectral library [33,34].

### 4.3. In Silico Predictions of Bioactivity and Pharmacokinetic Parameters (ADME)

For the in silico analyses, SMILES codes for selected GC–MS-detectable compounds in ESPA, namely n-hexadecanoic acid, oleic acid, and octadecanoic acid, were retrieved from the PubChem database https://pubchem.ncbi.nlm.nih.gov/ (accessed on 20 March 2026). PASSonline [35] was used as an exploratory bioactivity prediction tool to identify potential biological activities associated with each molecule, whereas SwissADME [36] was used to estimate selected pharmacokinetic and physicochemical parameters.

### 4.4. Molecular Docking

Molecular docking analysis was performed using AutoDock Vina Version 1.2.3 (Molecular Graphics Laboratory, The Scripps Research Institute, La Jolla, CA, USA), which is widely used to predict ligand–protein interactions and estimate binding affinities [37]. The three-dimensional structures of cyclooxygenase-1 (COX-1; PDB ID: 1CQE) and cyclooxygenase-2 (COX-2; PDB ID: 6COX) were obtained from the RCSB Protein Data Bank. These targets were selected because of their relevance to prostaglandin biosynthesis and the pharmacological mechanism of diclofenac.

Selected GC–MS-detectable compounds in ESPA, namely n-hexadecanoic acid, oleic acid, and octadecanoic acid, were used as ligands for docking analysis based on their reported anti-inflammatory potential. Diclofenac was included as a reference drug because of its established inhibitory activity against COX isoforms, allowing comparison with a therapeutic standard. Protein and ligand preparation was performed using formats compatible with AutoDock Vina. Non-structural water molecules were removed, appropriate charges were assigned, and ligand conformations were optimized prior to docking. The active site was defined using a grid box centered on the previously reported catalytic site of each COX isoform.

After defining the docking parameters, simulations were performed to generate multiple binding poses for each ligand. The resulting conformations were evaluated primarily according to their predicted binding affinity. The best-scored poses were further analyzed for hydrogen bonds, hydrophobic contacts, electrostatic interactions, and amino acid residues involved in ligand binding. These analyses were used to explore potential molecular interactions with COX-1 and COX-2, rather than to confirm direct enzymatic inhibition.

### 4.5. Animals

Male BALB/c mice weighing 25–30 g were obtained from the Animal Facility of the Division of Natural and Exact Sciences, University of Guanajuato. Animals were housed in a temperature-controlled room under a 12 h light/dark cycle (12:12 h), with free access to food and water. Experiments were conducted between 8:00 a.m. and 14:00 h to reduce variability associated with circadian rhythm.

All experimental procedures followed the Mexican Official Norm for the care and handling of laboratory animals (NOM-062-ZOO-1999) [38] and adhered to ethical guidelines for pain research in conscious animals [39] and the ARRIVE 2.0 guidelines https://arriveguidelines.org/ (accessed on 20 March 2026) [40]. The study was approved by the Internal Committee for the Care and Use of Laboratory Animals of the University of Guanajuato (CICUAL-P09-2025). Experimental procedures were designed and reported in accordance with ARRIVE 2.0 recommendations. Animals were randomly assigned to experimental groups using simple random allocation. Behavioral assessments were performed by an observer blinded to the treatment allocation. Each animal was used only once and allocated to a single experimental condition. The predefined primary outcome was the number of paw flinches recorded at fixed intervals during the formalin test. The number of animals per group was determined by statistical power analysis, and efforts were made to minimize animal use and suffering. At the end of the experiment, animals were euthanized immediately by cervical dislocation.

### 4.6. Acute Toxicity

The acute oral toxicity study of ESPA was conducted in accordance with the Organization for Economic Co-operation and Development (OECD) Guideline 423 for acute oral toxicity testing [41]. BALB/c mice received a single oral dose of ESPA (2000 mg/kg body weight; n = 3) dissolved in saline solution. Administration was performed by oral gavage using a 1 mL syringe fitted with a gavage probe. The selected dose corresponds to the limit dose recommended by OECD Guideline 423. After administration, each animal was housed individually and monitored at predefined intervals for mortality and clinical signs of toxicity, including changes in behavior, locomotor activity, posture, skin and mucous membranes, respiratory and cardiovascular function, central nervous system activity, and somatomotor responses. Mortality and clinical signs were evaluated at 10 min, 30 min, 1 h, 2 h, 4 h, and 6 h after administration. Thereafter, mortality was monitored twice daily, and clinical signs were assessed once daily for 14 days.

### 4.7. Experimental Design

The antinociceptive effects of ESPA (10, 30, 100, and 300 mg/kg, p.o.) and diclofenac (3, 10, 30, and 100 mg/kg, p.o.) were evaluated individually using the formalin test. In addition, four fixed-ratio combinations of ESPA and diclofenac were administered orally to assess their pharmacological interaction. Each experimental group included six mice, resulting in a total of 78 mice across 13 experimental groups. A vehicle group (saline solution; n = 6) was included as the control. Mice were assigned to the corresponding experimental groups using simple random allocation. The number of animals per group was calculated using G*Power 3.1.9.7 software based on statistical power analysis [42]. The calculation assumed an alpha error probability of 0.05, a statistical power of 0.80, 13 experimental groups, and an expected large effect size of f = 0.50. This effect size was selected a priori to detect robust pharmacological effects in the formalin test, where active antinociceptive treatments are expected to produce marked reductions in nociceptive behavior compared with vehicle-treated animals. Under these assumptions, a minimum sample size of six animals per group was considered sufficient to achieve adequate statistical power. Additionally, this decision was aligned with ethical principles for animal research (3Rs), particularly reduction, aiming to minimize animal use while maintaining experimental validity. Nevertheless, it is acknowledged that larger group sizes are frequently used in similar studies, and this aspect should be considered when interpreting the findings.

Drug or vehicle administration was performed 30 min before formalin injection [43]. The ED30 values for ESPA and diclofenac were estimated from their respective dose–response curves and subsequently used to evaluate the pharmacological interaction of the ESPA–diclofenac combination by isobolographic analysis.

### 4.8. Induction of the Pain Model

The formalin-induced nociception test was performed as previously described [43]. Mice were individually placed in a Plexiglas chamber for 30 min before testing to allow habituation. Subsequently, 20 µL of 2% formalin was injected subcutaneously into the dorsal surface of the right hind paw. Nociceptive behavior was quantified as the number of flinches of the formalin-injected paw, recorded for 1 min every 5 min over a 60 min observation period. The response was analyzed in two phases: phase I (0–10 min), corresponding to the neurogenic response, and phase II (15–60 min), corresponding predominantly to the inflammatory response. The area under the curve (AUC) for nociceptive behavior was calculated for each experimental group using the trapezoidal method, allowing integrated quantification of the response over time. The percentage of antinociception was then calculated from the AUC values using the following formula [44]:

(1) $$\%\;antinociception=\frac{AUC\;vehicle-UAC\;post\;compound}{AUC\;vehicle}\times 100$$

### 4.9. Isobologram

Isobolographic analysis was performed using the ED30 values obtained from the dose–response curves of ESPA and diclofenac administered individually and in combination. The isobologram was constructed by plotting the ED30 of ESPA on the *x*-axis and the ED30 of diclofenac on the *y*-axis. The line connecting the individual ED30 values represented the theoretical line of additivity. The theoretical additive ED30 (ED30T) for the fixed-ratio 1:1 combination was calculated from the individual ED30 values of ESPA and diclofenac and plotted on the additivity line [29].

Based on the individual ED30 values, four fixed-ratio 1:1 combinations were prepared and evaluated in the formalin test to generate the dose–response curve of the combination and calculate the experimental ED30 (ED30E). The ED30T and ED30E values were compared using Student’s *t*-test. The interaction index (γ) was calculated as described by Tallarida (2002) [45]. Values close to 1 indicate an additive interaction, values greater than 1 indicate antagonism or subadditivity, and values less than 1 indicate synergism or superadditivity.

(2) $$\gamma =\frac{ED_{30}\;of\;the\;combination\;(experimental)}{ED_{30}\;of\;the\;combination\;(theoretical)}$$

### 4.10. Statistical Analysis

Data are represented as mean ± standard deviation (SD). Before applying parametric tests, the assumptions of normality and homogeneity of variances were assessed. Normality was evaluated using the Shapiro–Wilk test, and homogeneity of variances was assessed using Brown–Forsythe test. Observations were considered independent because each animal was used only once and assigned to a single experimental group. When these assumptions were met, multiple comparisons were performed using one-way ANOVA followed by Dunnett’s post hoc test. When the assumptions were not met, non-parametric analysis was performed using the Kruskal–Wallis test followed by Dunn’s post hoc test. The statistical difference between the experimental ED30 (ED30E) and the theoretical additive ED30 (ED30T) was determined using Student’s *t*-test. Differences were considered statistically significant at *p* < 0.05. All statistical analyses were performed using GraphPad Prism 6.0 (GraphPad Software, San Diego, CA, USA).

## 5. Conclusions

In conclusion, ESPA produced a significant antinociceptive effect, particularly during the inflammatory phase of the formalin-induced pain model. Although diclofenac exhibited greater potency, consistent with its established cyclooxygenase-related pharmacological profile, ESPA showed consistent efficacy in reducing inflammatory nociceptive behavior. The biological activity of ESPA may be associated with its complex chemical composition, including inulin-type fructans previously described in the extract and long-chain fatty acids detected within the GC–MS-detectable fraction. However, because the present phytochemical characterization was mainly based on GC–MS, the specific constituents responsible for the observed antinociceptive activity cannot be definitively established. Therefore, the identified fatty acids should be interpreted as plausible contributors within a complex matrix rather than as confirmed active principles.

In silico predictions and molecular docking analyses provided preliminary, hypothesis-generating support for potential interactions with inflammatory pathways and COX isoforms, but these findings require experimental validation. Importantly, isobolographic analysis demonstrated a synergistic interaction between ESPA and diclofenac, suggesting a potential NSAID dose-sparing effect while preserving antinociceptive efficacy. The absence of acute oral toxicity at the OECD limit dose further supports the preliminary safety of ESPA under the tested conditions.

Importantly, the present findings should be interpreted within the limitations of the phytochemical characterization and mechanistic validation. The observed pharmacological effects cannot be attributed to specific individual constituents, and the proposed mechanisms are based on indirect and computational evidence. Therefore, ESPA should be considered as a complex phytochemical matrix whose biological activity arises from the combined interaction of multiple components rather than a single defined active compound.

## 6. Future Perspectives

Future studies should evaluate the long-term safety and repeated-dose toxicity of ESPA, as well as its efficacy in additional pain models, including chronic inflammatory and neuropathic pain. Pharmacokinetic and pharmacodynamic studies will also be necessary to characterize systemic exposure, identify bioavailable constituents, and optimize dosing regimens. Further phytochemical studies using LC–MS/MS-based metabolomic profiling, chromatographic fractionation, and bioassay-guided isolation should be performed to identify the compounds responsible for the observed pharmacological effects. In addition, direct biochemical assays, including COX activity assays and measurement of inflammatory mediators, are needed to validate the proposed mechanisms. From a translational perspective, the synergistic interaction observed between ESPA and diclofenac supports further investigation of combination strategies aimed at reducing NSAID exposure while maintaining antinociceptive efficacy. Such approaches may contribute to the development of safer multimodal strategies for inflammatory pain management, with ESPA as a potential pharmacological adjuvant rather than a stand-alone analgesic agent.

## Figures and Tables

**Table 1:** Compounds identified based on the matching of retention time (TR) in ESPA by GC-MS.

Peak	Retention Time	% Peak Area	Compound
1	3.61	11.35	Pentanal
2	4.9	5.30	3-Amino-2-oxazolidinone
3	5.21	3.95	Cyclohexan-1,4,5-triol-3-one-1-carboxylic acid
4	7.16	2.98	2,3-Anhydro-D-galactosan
5	8.23	8.54	trans-5-Ethyl-2,2-dimethylisoxazolidine
6	8.45	5.44	2-Formyl-9-(β-D-ribofuranosyl)hypoxanthine
7	8.92	15.36	Isobutyl N-(carbamoylmethyl)carbamate
8	10.2	2.97	2,4-Monoethylidene-L-xylitol
9	10.52	3.04	1,2-Hydrazinedicarboxamide
10	14.05	22.25	n-Hexadecanoic acid
11	15.88	8.01	Oleic acid
12	16.19	10.83	Octadecanoic acid

**Table 2:** Predicted ADME/T (absorption, distribution, metabolism, excretion, and toxicity) properties for long-chain fatty acid compounds of ESPA.

	Parameter									
Compound	No. H-Bond Donors	No. H-Bond Acceptors	TPSA Å^2^	Log Po/w (MLOGP)	Log S (ESOL)	Solubility	GI Absorption	Pains	Lipinski Filter (No. Violations)	CYP2C9 Inhibitor
**n-Hexadecanoic acid**	1	2	37.30	5.55	−5.02	Moderately soluble	High	0	1	Yes
**Octadecanoic acid**	1	2	37.30	6.33	−5.73	Moderately soluble	High	0	1	No
**Oleic acid**	1	2	37.30	6.11	−5.41	Moderately soluble	High	0	1	Yes

**Table 3:** Pharmacological activities predicted for the main ESPA compounds using PASSonline.

Compound	Prostaglandin-A1 DELTA-Isomerase Inhibitor		Leukotriene-B4 20-Monooxygenase Inhibitor		Prostaglandin-E2 9-Reductase Inhibitor		Thromboxane B2 Antagonist		TNF Expression Inhibitor		Leukotriene-C4 Synthase Inhibitor		15-Hydroxyprostaglandin-D dehydrogenase (NADP+) Inhibitor	
	Pa	Pi	Pa	Pi	Pa	Pi	Pa	Pi	Pa	Pi	Pa	Pi	Pa	Pi
**n-Hexadecanoic acid**	0.921	0.001	0.860	0.002	0.841	0.008	0.728	0.003	--	--	--	--	--	--
**Octadecanoic acid**	0.921	0.001	0.860	0.02	0.841	0.0089	0.728	0.002	--	--	--	--	0.755	0.002
**Oleic acid**	0.891	0.002	0.90	0.001	0.924	0.004	--	--	0.724	0.005	0.720	0.001	0.854	0.001

**Table 4:** Molecular docking analysis of hexadecanoic, octadecanoic, and oleic acids in COX-1 and COX-2.

Ligand	Protein	ΔG (kcal/mol)	Ki (µM)	Interaction Type	Interacting Residues
**Diclofenac**	COX-1	−6.330	22.71	1 H-bond + hydrophobic interactions	Tyr355A, Leu112A, Val116A, Leu115A, Leu93A
	COX-2	−8.202	0.96	Hydrophobic/aromatic interactions	Tyr355A, Val349A, Ala527A, Trp387A
**n-Hexadecanoic acid**	COX-1	−6.116	32.60	Ionic interaction with Arg + hydrophobic interactions	Arg581A, Phe580A, Gly354A, Gln351A, Gln350A, Glu347A, Arg581A
	COX-2	−6.123	32.22	2 H-bond + hydrophobic interactions	Ser530A, Tyr385A, Leu352A, Val349A, Leu359A, Leu531A, Val523A, Val116A, Ala527A, Gly526A, Tyr355A, Trp387A, Phe518A
**Octadecanoic acid**	COX-1	−6.296	24.06	1 H-bond + hydrophobic interactions	Glu524A, Thr89A, Val116A, Leu115A, Leu93A
	COX-2	−5.021	207.28	1 H-bond + hydrophobic interactions	Glu524A, Val89A, Ile92A, Tyr115A, Leu93A, Val116A
**Oleic acid**	COX-1	−5.698	66.06	Ionic interaction with Arg + hydrophobic interactions	Tyr385A, Tyr355A, Val116A, Gly526A, ILE523A, LEU93A, Ala527A, Leu352A
	COX-2	−5.370	114.96	2 H-bond + hydrophobic interactions	Ser530A, Tyr385A, Val116A, Val349A, Leu352A, Ala527A, Gly526A, Tyr355A, Trp387A

**Table 5:** Diclofenac and ESPA doses evaluated in combination at a 1:1 ratio.

Combination	Dose (mg/kg)		
	Diclofenac	ESPA	Total
ED_30_ + ED_30_	1.37	34.47	35.84
(ED_30_ + ED_30_)/2	0.68	17.24	17.92
(ED_30_ + ED_30_)/4	0.34	8.62	8.96
(ED_30_ + ED_30_)/8	0.17	4.31	4.48

## Data Availability

The original contributions presented in this study are included in the article. Further inquiries can be directed to the corresponding authors.

## References

[1] Hakim S., Jain A., Woolf C.J. (2024). Immune drivers of pain resolution and protection. Nat. Immunol..

[2] Jain A., Hakim S., Woolf C.J. (2024). Immune drivers of physiological and pathological pain. J. Exp. Med..

[3] Ji R.-R., Huh Y., Ji Y. (2023). Inflammatory mediators, nociceptors, and their interactions in pain. Neuroimmune Interact. Pain..

[4] Vane J.R., Botting R.M. (1998). Mechanism of action of nonsteroidal anti-inflammatory drugs. Am. J. Med..

[5] Bindu S., Mazumder S., Bandyopadhyay U. (2020). Non-steroidal anti-inflammatory drugs (NSAIDs) and organ damage: A current perspective. Biochem. Pharmacol..

[6] LaForge J.M., Urso K., Day J.M., Bourgeois C.W., Ross M.M., Ahmadzadeh S., Cornett E.M., Kaye A.D. (2023). Non-steroidal anti-inflammatory drugs: Clinical implications, renal impairment risks, and acute kidney injury. Adv. Ther..

[7] Moudgil K.D., Venkatesha S.H. (2023). The anti-inflammatory and immunomodulatory activities of natural products to control autoimmune inflammation. Int. J. Mol. Sci..

[8] Patel M., Wahezi S., Mavrocordatos P., Abd-Elsayed A. (2025). The effects and mechanisms of phytochemicals on pain management and analgesic therapy. Nutrients.

[9] Bermúdez-Bazán M., Castillo-Herrera G.A., Urias-Silvas J.E., Escobedo-Reyes A., Estarrón-Espinosa M. (2021). Hunting bioactive molecules from the *Agave* genus: An update on extraction and biological potential. Molecules.

[10] Pérez-López A.V., Simpson J., Clench M.R., Gomez-Vargas A.D., Ordaz-Ortiz J.J. (2021). Localization and composition of fructans in stem and rhizome of *Agave tequilana* Weber var. azul. Front. Plant Sci..

[11] El-Hawary S.S., El-Kammar H.A., Farag M.A., Saleh D.O., El Dine R.S. (2020). Metabolomic Profiling of Five Agave Leaf Taxa via UHPLC/PDA/ESI-MS in Relation to Their Anti-Inflammatory, Immunomodulatory and Ulceroprotective Activities. Steroids.

[12] Gutiérrez-Nava Z.J., Jiménez-Aparicio A.R., Herrera-Ruiz M.L., Jiménez-Ferrer E. (2017). Immunomodulatory effect of *Agave tequilana* evaluated on an autoimmunity like-SLE model induced in Balb/c mice with pristane. Molecules.

[13] Santa-María C., López-Enríquez S., Montserrat-de la Paz S., Geniz I., Reyes-Quiroz M.E., Moreno M., Palomares F., Sobrino F., Alba G. (2023). Update on anti-inflammatory molecular mechanisms induced by oleic acid. Nutrients.

[14] Zhang S., Roth B.L. (2023). Sensing unsaturated fatty acids: Insights from GPR120 signaling. Cell Res..

[15] Pan Y., Ren H., Lan L., Li Y., Huang T. (2023). Review of predicting synergistic drug combinations. Life.

[16] Yamamoto T., Nozaki-Taguchi N. (2002). The role of cyclooxygenase-1 and -2 in the rat formalin test. Anesth. Analg..

[17] Costa L.T.S.d., Fracasso J.A.R., Guarnier L.P., Brito G.R.d., Fumis D.B., Camargo Bittencourt R.A.d., Guiotti A.M., Barros Barbosa D.d., Camargo I.C.C., Souza E.B.d. (2023). Toxicity and Anti-Inflammatory Effects of Agave sisalana Extract Derived from Agroindustrial Residue. Plants.

[18] Monterrosas-Brisson N., Ocampo M.L., Jiménez-Ferrer E., Jiménez-Aparicio A.R., Zamilpa A., Gonzalez-Cortazar M., Tortoriello J., Herrera-Ruiz M. (2013). Anti-inflammatory activity of different Agave plants and the compound cantalasaponin-1. Molecules.

[19] Dunder R.J., Quaglio A.E.V., Maciel R.P., Luiz-Ferreira A., Almeida A.C.A., Takayama C., Faria F.M., Souza-Brito A.R.M. (2010). Anti-inflammatory and analgesic potential of hydrolyzed extract of Agave sisalana Perrine ex Engelm., Asparagaceae. Rev. Bras. Farmacogn..

[20] Resino-Ruiz D., Gonzalez-Madariaga Y., Nieto L., Linares Y.M., León J.O.G., Martín A.V., Díaz A.V., Torrens F., Castillo-Garit J.A. (2023). Anti-inflammatory activity: In silico and in vivo of sapogenins present in Agave brittoniana subsp. brachypus (Trel.). Anti-Inflamm. Anti-Allergy Agents Med. Chem..

[21] Castañeda-Corral G., Cedillo-Cortezano M., Aviles-Flores M., López-Castillo M., Acevedo-Fernández J.J., Petricevich V.L. (2024). Antinociceptive and anti-inflammatory activities of acetonic extract from Bougainvillea x buttiana (var. Rose). Pharmaceuticals.

[22] Ríos-Carlos M., Jiménez M., Cervantes-García D., Córdova-Dávalos L.E., Verduzco L.E., Enríquez-Medrano F.J., Fa-bela-Sánchez O., Bermúdez-Humarán L.G., Salinas E. (2025). Immunomodulatory and Anti-Inflammatory Effects of Agave Fructans in Atopic Dermatitis: Gut Microbiota and Short-Chain Fatty Acid Implication. Front. Immunol..

[23] Calder P.C. (2012). Fatty acids and inflammation: The cutting edge between food and pharma. Eur. J. Pharmacol..

[24] Hidalgo M.A., Carretta M.D., Burgos R.A. (2021). Long chain fatty acids as modulators of immune cells function: Contribution of FFA1 and FFA4 receptors. Front. Physiol..

[25] Lorente-Cebrián S., Costa A.G.V., Navas-Carretero S., Zabala M., Martínez J.A., Moreno-Aliaga M.J. (2013). Role of omega-3 fatty acids in obesity, metabolic syndrome, and cardiovascular diseases: A review of the evidence. J. Physiol. Biochem..

[26] Calder P.C. (2010). Omega-3 fatty acids and inflammatory processes. Nutrients.

[27] Hughes R.L., Alvarado D.A., Swanson K.S., Holscher H.D. (2022). The Prebiotic Potential of Inulin-Type Fructans: A Systematic Review. Adv. Nutr..

[28] Farabegoli F., Santaclara F.J., Costas D., Alonso M., Abril A.G., Espiñeira M., Ortea I., Costas C. (2023). Exploring the Anti-Inflammatory Effect of Inulin by Integrating Transcriptomic and Proteomic Analyses in a Murine Macrophage Cell Model. Nutrients.

[29] Tallarida R.J. (2001). Drug synergism: Its detection and applications. J. Pharmacol. Exp. Ther..

[30] Łuszczki J.J., Wlaź P. (2023). Isobolographic analysis of drug combinations: Theory and practice. Pharmacol. Rep..

[31] Walker C., Biasucci L.M. (2018). Cardiovascular safety of non-steroidal anti-inflammatory drugs. Eur. Heart J..

[32] Verma D.K., Patel A.R., Thakur M., Singh S., Tripathy S., Srivastav P.P., Chávez-González M.L., Gupta A.K., Aguilar C.N. (2021). A review of the composition and toxicology of fructans, and their applications in foods and health. J. Food Compos. Anal..

[33] Sani M.S.A., Bakar J., Abdul Rahman R., Abas F. (2020). Effects of coated capillary column, derivatization, and temperature programming on the identification of Carica papaya seed extract composition using GC–MS analysis. J. Anal. Test..

[34] Stein S.E. (2012). Mass spectral reference libraries: An ever-expanding resource for chemical identification. Anal. Chem..

[35] Filimonov D.A., Lagunin A.A., Gloriozova T.A., Rudik A.V., Druzhilovskii D.S., Pogodin P.V., Poroikov V.V. (2014). Prediction of the biological activity spectra of organic compounds using the PASS online web resource. Chem. Heterocycl. Compd..

[36] Daina A., Michielin O., Zoete V. (2017). SwissADME: A free web tool to evaluate pharmacokinetics, drug-likeness and medicinal chemistry friendliness of small molecules. Sci. Rep..

[37] Trott O., Olson A.J. (2010). AutoDock Vina: Improving the speed and accuracy of docking with a new scoring function, efficient optimization, and multithreading. J. Comput. Chem..

[38] (1999). Secretariat of Agriculture, Livestock, Rural Development, Fisheries and Food (SAGARPA). NOM-062-ZOO-1999. Technical Specifications for the Production, Care, and Use of Laboratory Animals. Official Journal of the Federation (Diario Oficial de la Federación). https://www.researchgate.net/publication/11280294_Laboratory_animals_and_official_Mexican_norm_NOM-062-ZOO-1999.

[39] Zimmermann M. (1983). Ethical guidelines for investigations of experimental pain in conscious animals. Pain.

[40] Percie du Sert N., Hurst V., Ahluwalia A., Alam S., Avey M.T., Baker M., Browne W.J., Clark A., Cuthill I.C., Dirnagl U. (2020). The ARRIVE guidelines 2.0: Updated guidelines for reporting animal research. PLoS Biol..

[41] Organisation for Economic Co-Operation and Development (OECD) (2002). OECD Guideline for the Testing of Chemicals No. 423: Acute Oral Toxicity—Acute Toxic Class Method.

[42] Bate S.T., Clark R.A. (2014). The Design and Statistical Analysis of Animal Experiments.

[43] Dubuisson D., Dennis S.G. (1977). The formalin test: A quantitative study of the analgesic effects of morphine, meperidine, and brain stem stimulation in rats and cats. Pain.

[44] Gibaldi M. (1991). Biopharmaceutics and Clinical Pharmacokinetics.

[45] Tallarida R.J. (2002). The interaction index: A measure of drug synergism. Pain.

